# Micro-supercapacitors based on oriented coordination polymer thin films for AC line-filtering†

**DOI:** 10.1039/c8ra06474a

**Published:** 2018-08-30

**Authors:** Weiwei Hua, Jingwei Xiu, Fei Xiu, Zepu Zhang, Juqing Liu, Linfei Lai, Wei Huang

**Affiliations:** Key Laboratory of Flexible Electronics (KLOFE), Institute of Advanced Materials (IAM), Jiangsu National Synergetic Innovation Center for Advanced Materials (SICAM), Nanjing Tech University (Nanjing Tech) 30 South Puzhu Road Nanjing 211816 P. R. China Iamfxiu@njtech.edu.cn iamlflai@njtech.edu.cn; Shanxi Institute of Flexible Electronics (SIFE), Northwestern Polytechnical University (NPU) 127 West Youyi Road Xi'an 710072 P. R. China iamwhuang@njtech.edu.cn; Department of Materials Science and Engineering, Southern University of Science and Technology Shenzhen Guangdong P. R. China

## Abstract

Reported herein is a facile solution-processed substrate-independent approach for preparation of oriented coordination polymer (Co-BTA) thin-film electrodes for on-chip micro-supercapacitors (MSCs). The Co-BTA-MSCs exhibited excellent AC line-filtering performance with an extremely short resistance–capacitance constant, making it capable of replacing aluminum electrolytic capacitors for AC line-filtering applications.

## Abstract 2

Micro-supercapacitors exhibiting excellent AC line-filtering with oriented coordination polymer thin-film electrodes are fabricated based on a substrate-independent electrode fabrication strategy.

## Graphical abstract

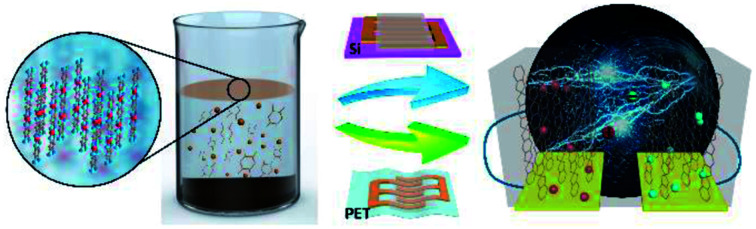

Micro-supercapacitors (MSCs), as important Si-compatible on-chip electrochemical energy storage devices, have attracted rapidly growing attention due to their rapid energy-harvesting features and burst-mode power delivery.^1,2^ In the past few years, a variety of materials including carbon nanotubes,^3^ graphene,^4^ graphene oxide and mesoporous conducting polymers,^5,6^ have already been explored to fabricate the electrodes of MSCs for improving their electrochemical performance. Unfortunately, fabrication procedures of most of these active materials suffer from high cost, harsh and complicated processing conditions, as well as easy cracking and delamination of active films,^1,4^ extremely limiting their commercial applications. Moreover, their performances are unsatisfactory for alternating current (AC) line-filtering, which is a key parameter to implement high-frequency operation in most line-powered devices.^7–9^

For AC line-filtering, capacitors need to respond harmonically at 120 Hz to attenuate the leftover AC ripples on direct current voltage busses.^10^ Notably, the development of more compact and miniaturized capacitors to replace traditional aluminum electrolytic capacitors (AECs) for AC line-filtering has become one of the major tasks for future electronics.^11^ However, typical supercapacitors are incapable for AC line-filtering at this frequency due to their limited ion diffusion and charge transfer efficiency, corresponding to the unsuitable architectures and low conductivity of electrode materials.^10–12^ Therefore, the design and fabrication of highly conductive electrodes with optimized architectures for facial electron/ion transportation is crucial for improving the performance of MSCs in AC line-filtering.^12,13^ It is worth mentioning that great advancements have been achieved by utilizing vertically oriented graphene sheets as well as 3-dimensional graphene/carbon nanotube carpets prepared by chemical vapor deposition (CVD),^7,8^ yielding efficient filtering of 120 Hz AC with short resistance–capacitance (RC) time constants of less than 0.2 ms, which is competitive with those of porous carbon-based supercapacitors (RC time constant = 1 s) as well as AECs (RC time constant = 8.3 ms).^8^ However, the CVD method necessitated in the fabrication of graphene/carbon nanotube electrodes suffers from high cost and complicate procedures.

Coordination polymers with an unrivalled degree of structural and property tunability which could be realized by facial procedures, are promising candidates for energy storage.^14,15^ Recently, a remarkable achievement which demonstrated a facile and low-cost solution-processed method towards on-chip MSCs based on an azulene-bridged coordination polymer framework (PiCBA) on a Si wafer-supported Au surface was reported.^14^ Nevertheless, the reported preparation of coordination polymer film exhibited strong dependence on the surface chemistry (functionality) of the substrate and further improvement of their electrochemical stability was needed. Therefore, the development of substrate-independent fabrication strategies of large-scale and uniform coordination polymer films is in great need not only for fundamental studies, but also for technological applications especially in electronics.

Herein, we demonstrate a facial solution-based substrate-independent approach to fabricate oriented coordination polymer (Co-BTA) thin-film electrodes. Remarkably, rigid and flexible Co-BTA-based MSCs with excellent electrochemical stability and AC line-filtering performance were realized, indicating great application potential in micro-supercapacitors.

As demonstrated in Fig. 1a–c, a large scale and continuous Co-BTA coordination polymer film composed of one dimensional (1D) molecules ([Co(1,2,4,5-bta)]_*n*_) was prepared at the air–liquid interface through a coordination reaction between 1,2,4,5-benzenetetramine tetrahydrochloride (BTA) and cobalt acetate tetrahydrate (Co(CH_3_COO)_2_·4H_2_O). Notably, the preparation of Co-BTA film is basing on mild conditions and independent of any substrates. The resulting film can be transferred onto any supports including rigid silicon (Si) wafer, glass, as well as flexible PET substrate, indicating great substrate-independence and making it practically applicable for various applications. Besides of a brown film formed at the air–liquid interface, a powder product is also obtained at the bottom of the reaction bottle.

**Fig. 1:**
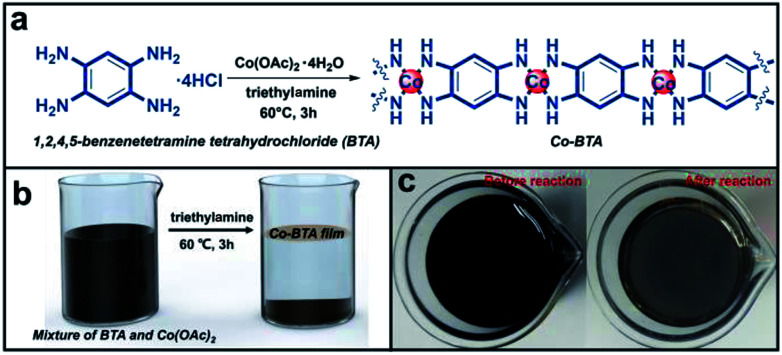
(a) Synthesis of Co-BTA through the coordination reaction between BTA and cobalt ions. (b) Illustration of the gas–liquid interface growth of Co-BTA film. (c) Photographs of the reaction system before and after the coordination reaction.

To study the morphology of the resulting Co-BTA film, the brown film was transferred onto a SiO_2_/Si wafer by immersing the wafer down to the reaction mixture and subsequently lifting the film up. The scanning electron microscopy (SEM) image reveals a highly uniform and large-scale distribution of the obtained film without cracks or wrinkles (Fig. 2a), which is superior to other reported coordination polymer films obtained *via* a similar method.^16^ An average thickness of approximately 60 nm of the Co-BTA film is observed from the cross-sectional SEM image as shown in Fig. 2b. Interestingly, thickness of the obtained coordination polymer film could be well controlled and Co-BTA films with thicknesses up to several hundred nanometers could be well prepared by adjusting the ratio of raw materials (Fig. 2c and d).

**Fig. 2:**
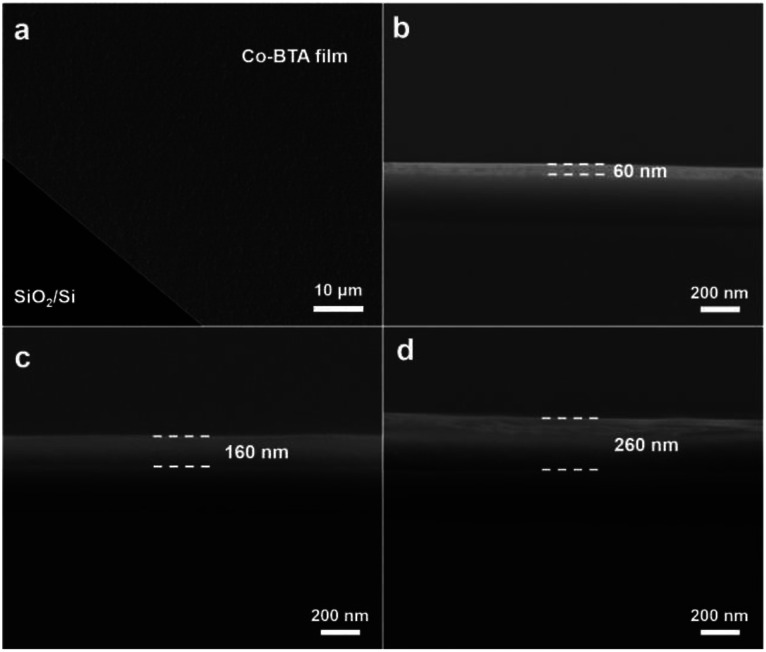
(a) Planar SEM image of Co-BTA film. Cross-sectional SEM images of Co-BTA films with a thickness of (b) 60 nm, (c) 160 nm and (d) 260 nm.

To investigate the structure information of the resulting Co-BTA and further explore the coordination reaction, characterizations including powder X-ray diffraction measurements (PXRD), X-ray photoelectron spectroscopy (XPS) and Fourier transform infrared spectroscopy (FTIR) were carried out. The PXRD pattern of Co-BTA powder shown in Fig. S1a† is in great agreement with that simulated from the crystal structure of Ni(dhbq)·*n*H_2_O (Fig. S1b†), suggesting that Co-BTA and Ni(dhbq)·*n*H_2_O is isostructural and forms 1D structures with straight infinite chain extends.^17^ More interestingly, PXRD measurements employing two different scattering geometries (Fig. S2†) on the Co-BTA thin-film demonstrate two quite different diffraction patterns. As observed in Fig. 3a, the diffraction pattern observed for the out-of-plane scattering geometry exhibits three characteristic peaks of the Co-BTA film at ∼12°, 24° and 36°, which are corresponding to (001), (002) and (003), respectively. In contrast, the in-plane PXRD profile employing grazing-incidence XRD (GIXRD) technique at an incident angle (*α*) of 0.2° demonstrates a main peak at ∼18°, which is assigned to the (110) diffraction peak. Different diffraction peaks observed through these two XRD scattering geometries indicate an orientation nature of the as-prepared Co-BTA film,^18^ which exhibits better crystallinity compared with the powder Co-BTA product. In addition, the N 1s core level spectrum for Co-BTA film exhibit one typical peak at 399.1 eV, which is corresponding to the amido coordinated with Co^II^, indicating the strong coordination between Co^II^ and BTA (Fig. 3b). The weak peak at ∼401 eV is assigned to N–O due to the oxidation of ligand BTA in ambient environment before reaction. The atomic ratio of N : Co is calculated to be 3.53 : 1 for Co-BTA film and 3.71 : 1 for Co-BTA powder respectively (Fig. S3 and Table S1†), which is close to the theoretical stoichiometric ratio (4 : 1) for Co-BTA structure, suggesting a high degree of coordination in the resulting product through one Co cation and two benzenetetramine groups. Moreover, the disappearance of two characteristic N–H stretching modes from –NH_2_ after the coordination reaction whereas the phenyl-related vibration still exists, further confirms the existence of –NH– in the product through the loss of one H per –NH_2_ (Fig. S4†).^19^

**Fig. 3:**
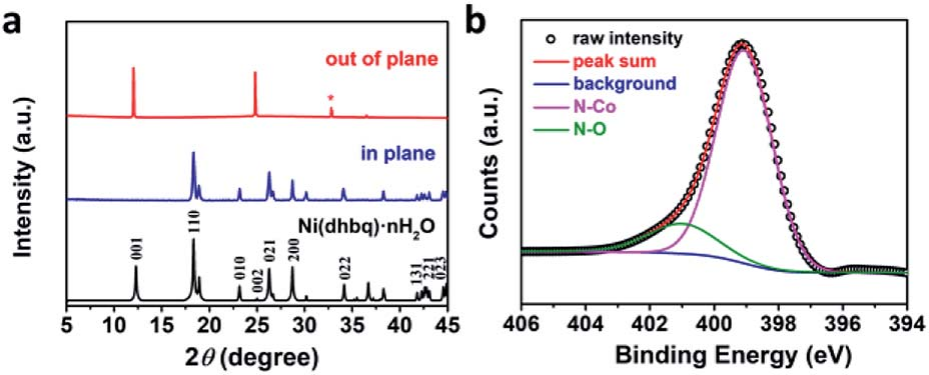
(a) PXRD profiles of out-of-plane XRD, in-plane XRD and simulated PXRD pattern of Ni(dhbq)·*n*H_2_O,^17^ respectively. *SiO_2_/Si substrate. (b) N 1s core level spectra of the Co-BTA film.

On the basis of facial fabrication, substrate independence, highly orientation nature, low band gap (1.68 eV, calculated from Fig. S5†) and excellent stability in acid environment (Fig. S6†), the resulting Co-BTA film is considered as a promising candidate for MSCs application. Fig. 4a schematically depicts the stepwise fabrication of a planar Co-BTA film based MSC on a SiO_2_/Si wafer and its electrochemical performance is first examined by cyclic voltammetry (CV) with scan rates ranging from 50 mV s^−1^ to 1000 V s^−1^ (Fig. 4b and c). At a low scan rate of 50 mV s^−1^, the 60 nm-thick Co-BTA film based MSC exhibited a pronounced pseudocapacitive effect, implying the occurance of faradaic reaction.^20^ With the increase of scan rate, a gradual transition of the CV curves from the pseudocapacitive to the typical electrical double-layer capacitive behavior with a nearly rectangular CV shape was observed. Remarkably, the device exhibited a maximum volumetric capacitance of 23.1 F cm^−3^ at 50 mV s^−1^, which is comparable with those of reported carbon- or graphene-based MSCs (Table S2†), *e.g.*, onion-like carbon,^21^ vertically oriented graphene,^8^ and carbon nanotubes/graphene.^7^ Even though a trend that *C*_V_ decreased gradually with increasing scan rate was observed, the Co-BTA-based electrode still delivered a *C*_V_ of 2.7 F cm^−3^ even at a high scan rate of 1000 V s^−1^, suggesting an excellent capacitive performance of this Co-BTA-based MSC device.^7^

**Fig. 4:**
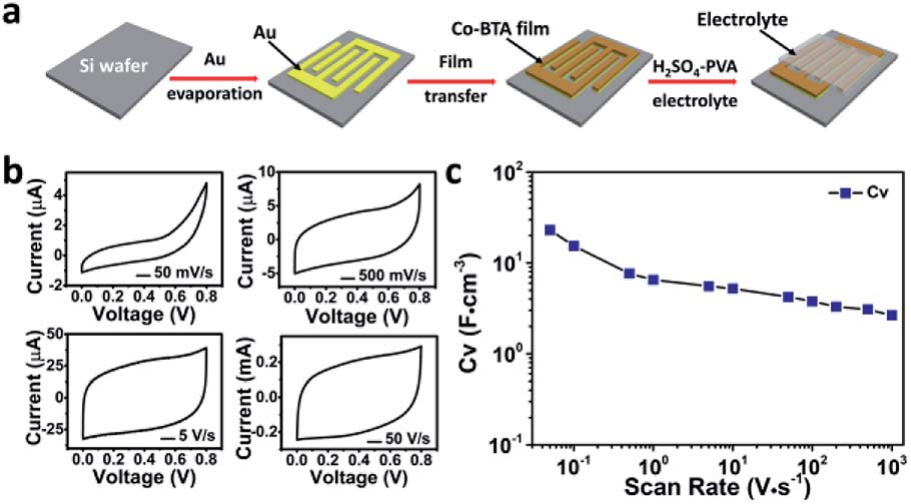
(a) Schematic illustration of the fabrication of MSC device with the Co-BTA film electrode. (b) CV curves of Co-BTA-based MSCs in the H_2_SO_4_–PVA gel electrolyte at different scan rates. (c) *C*_V_ evolution of the MSCs at different scan rates.

Electrochemical impedance spectroscopy (EIS) measurements were performed to evaluate the charge transport properties within the Co-BTA-based MSCs. The Nyquist plot shown in Fig. S7† indicated the kinetic features of electron transfer/ion diffusion at the electrode, from which an almost straight line especially in the low frequency region is observed. Notably, the plot shows a closed 90° slope without a charge transport semicircle at high frequency which is corresponding to an almost ideal capacitive ion diffusion mechanism, due to the excellent charge transfer property of the oriented Co-BTA electrode film. Moreover, this microdevice exhibited a low equivalent series resistance of 13.48 Ω (Fig. S7† (inset)), further suggesting the ultrafast ion diffusion characteristic in such a Co-BTA-based-MSC.^22^ It's suggested that the unique kinetic feature of fast ion diffusion and charge transfer benefits from the intrinsic characteristics of the oriented polymer film composed of 1D molecules, which can not only facilitate rapid ionic diffusion but also facilitate the interfacial charge transfer and faradaic redox reaction between the electrode material and electrolyte.

What's more, the dependence of the phase angle on frequency shown in Fig. 5a delivered a high characteristic frequency *f*_0_ of 6812 Hz at the phase angle of −45° (the resistance and reactance of the device have equal magnitudes),^10^ which is much higher than that of an active carbon supercapacitor (5 Hz),^23^ sulfur-doped graphene MSCs (3836 Hz),^22^ or an azulene-bridged coordination polymer framework based MSCs (PiCBA-MSCs) (3620 Hz) and so on,^14^ as summarized in Table S2.† Moreover, a max phase angle of −80° at a frequency of 18 Hz is observed, indicating the performance of this Co-BTA based MSCs is 89% of that of an ideal capacitor. Importantly, a large impedance phase angle of −78.6° was obtained at a frequency of 120 Hz, which is the largest reported value for coordination polymer based MSCs (Table S2†), suggesting an excellent AC line-filtering performance of our microdevice.^7^ To further conform the ultrahigh fast ion diffusion in Co-BTA-based-MSCs, the relaxation time constant *τ*_0_ (*τ*_0_ = 1/*f*_0_, the minimum time needed to discharge all the energy from the device with an efficiency of greater than 50% of its max. value) was calculated to be only 0.15 ms (6812 Hz), which is orders of magnitude higher than that of conventional electrical double-layer capacitors (1 s),^8^ activated or onion-like carbon MSCs (<200 ms, <10 ms),^21,23^ and much shorter than those of MSCs based on carbon nanotubes/reduced graphene oxide (4.8 ms) as well as azulene-bridged PiCBA coordination polymer framework film (0.27 ms).^14,24^ Moreover, a short RC time constant (*τ*_RC_) of 0.32 ms was obtained (Fig. 5b) through a series-RC circuit model, making it capable of replacing AECs for AC line-filtering application. To the best of our knowledge, this is the first report of coordination polymer-based MSCs exhibiting such a small relaxation time constant and RC time constant.

**Fig. 5:**
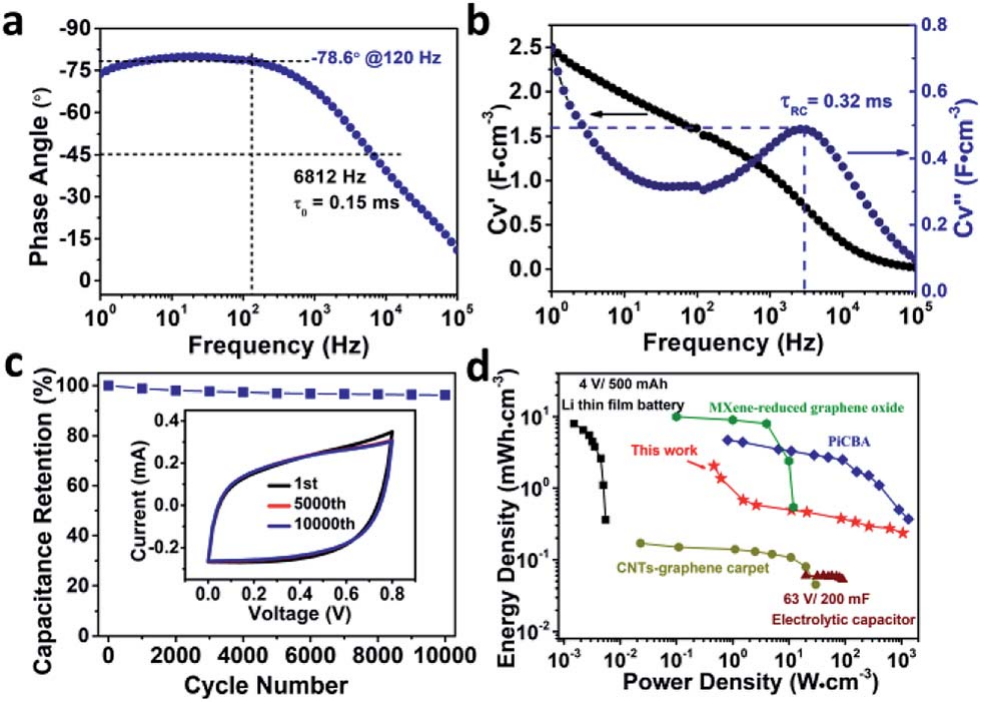
(a) Impedance phase angle on the frequency for the Co-BTA-based microdevices. (b) Plot of capacitance (*C*_V_′ = volumetric real capacitance and *C*_V_′′ = imaginary capacitance) *versus* the frequency of Co-BTA-based microdevices. (c) Cycling stability of Co-BTA film with 10 000 cycles at the scan rate of 50 V s^−1^. Inset displays the CV curves at the first, five thousandth and ten thousandth cycle, respectively. (d) Ragone plots for Co-BTA film, compared with commercially applied Li-thin-film batteries,^21^ electrolytic capacitors,^2^ CNT-graphene carpets,^24^ PiCBA coordination polymer and MXene-reduced graphene oxide.^14,25^

Impressively, this oriented electrode structure exhibits excellent long-term electrochemical stability with ∼96.3% capacitance retention even after 10 000 cycles of charging/discharging at a scan rate of 50 V s^−1^ (Fig. 5c), which has also been confirmed by comparing the CV curves before and after testing for 10 000 cycles (inset of Fig. 5c). It's worth pointing out that the as-made Co-BTA-based MSCs exhibit the best electrochemical stability among reported MSCs with coordination polymer electrodes.^14^ On the basis of the above discussion, it is reasonable to conclude that the ultrahigh fast ion diffusion/charge transfer in Co-BTA-based-MSCs attributed to the oriented architecture of Co-BTA thin-film electrodes, the excellent AC line-filtering performance, as well as remarkable electrochemical stability contributes to the excellent performances of Co-BTA-based-MSCs. Moreover, the power density and energy density of the as-made device is calculated and compared with that of MSCs based on other electrode materials to evaluate the energy storage performance of the Co-BTA based MSCs. The Ragone plot in Fig. 5d reveals a high power density of 1056 W cm^−3^ for our device, which is at least five orders of magnitude higher than that of commercial thin-film lithium batteries. What's more, our device exhibits an energy density of up to 1.6 mW h cm^−3^ at 50 mV s^−1^, which is at least one order of magnitude higher than that obtained for MSCs based on CNTs-graphene carpet and high-power electrolytic capacitors.^2,24^

To further demonstrate the substrate independence of this fabrication strategy, flexible Co-BTA-based-MSC device was fabricated and investigated basing on a flexible polyethylene terephthalate (PET) substrate instead of rigid Si substrate in the same way (Fig. S8–S10†). The as-fabricated device exhibited a maximum volumetric capacitance of 22.0 F cm^−3^ at 50 mV s^−1^, a short relaxation time constant *τ*_0_ of 0.15 ms and a RC time constant (*τ*_RC_) of 0.42 ms, which are close to the properties of devices with a Si substrate, confirming the substrate independence of this fabrication scheme. As a proof-of-concept application, bending tests were carried out and the bended device (radius = 1 cm) exhibited a small relaxation time constant *τ*_0_ of 0.21 ms and RC time constant (*τ*_RC_) of 0.42 ms, suggesting that the Co-BTA-based MSC with PET substrate in a bended state still delivers a good ion diffusion and AC line-filtering performance.

In conclusion, we have demonstrated a facile method that can be used to construct large scale and highly oriented uniform Co-BTA coordination polymer thin films using a very convenient and fast process. With this method, Co-BTA-based MSCs are fabricated without any dependence of the substrate. The as-fabricated MSCs on Si substrate exhibit high specific capacitance, energy density as well as excellent electrochemical stability. Particularly, the fabricated Co-BTA based MSCs deliver excellent AC line-filtering performance with an extremely short RC time of 0.32 ms, attributed to the facilitated ion diffusion beneficial from the oriented architecture of Co-BTA thin film. The high-performance electrochemical properties of Co-BTA-MSCs makes Co-BTA films promising materials to provide more compact AC filtering units for future electronic devices.

## Conflicts of interest

The authors declare no conflict of interest.

## Supplementary Material

RA-008-C8RA06474A-s001
