# Supplementary material for: Micro-supercapacitors based on oriented coordination polymer thin films for AC line-filtering
Source: RSC Adv. 2018 Aug 30;8(53):30624–8. doi: 10.1039/c8ra06474a (PMC9085491; doi:10.1039/c8ra06474a)
Supplement: RA-008-C8RA06474A-s001 [file RA-008-C8RA06474A-s001.pdf]

Electronic Supplementary Material (ESI) for RSC Advances.
This journal is © The Royal Society of Chemistry 2018

Electronic Supplementary Information
For

Micro-Supercapacitors Based on Oriented Coordination Polymer Thin Films for AC Line-Filtering

Weiwei Hua[a1], Jingwei Xiu[c1], Fei Xiu*[a1], Zepu Zhang[a], Juqing Liu[a], Linfei Lai*[a] and Wei Huang*[ab]

[a]Key Laboratory of Flexible Electronics (KLOFE) & Institute of Advanced Materials (IAM), Jiangsu National Synergetic Innovation Center for Advanced Materials (SICAM), Nanjing Tech University (NanjingTech), 30 South Puzhu Road, Nanjing 211816, P. R. China.

[b]Shanxi Institute of Flexible Electronics (SIFE), Northwestern Polytechnical University (NPU), 127 West Youyi Road, Xi'an 710072, P. R.China.

[c]Department of Materials Science and Engineering, Southern University of Science and Technology, Shenzhen, Guangdong, P. R. China.

*Corresponding author: iamfxiu@njtech.edu.cn;

iamlflai@njtech.edu.cn;

iamwhuang@njtech.edu.cn

Experimental section

Synthesis of Co-BTA: A solution of 80 mg (0.28 mmol) of benzene-1,2,4,5-tetrayltetraamine tetrahydrochloride purchased from Beijing Huaweiruike Chemical Co.,Ltd in 20 mL of water and 1120 μL of triethylymine (1.4 mol L^{-1}) was added to a solution of 26 mg (0.11 mmol) of cobalt acetate tetrahydrate ($Co(CH_3COO)_2{\bullet}4H_2O$) in 20 mL of water. The mixture was stirred in a bottle and kept at 60 °C for 3 hours, leading to the formation of a brown film at the gas-

[1] These authors contributed equally to this work.

liquid interface and deep brown powders at the bottom of the reaction bottle. The resulting film product was rinsed with ethonal and deionized (DI) water, finally transferred onto certain substrates and dried under vacuum at 60 ℃ for further characterizations and applications. Meanwhile, the powder product was collected, centrifuged, filtered, and then washed with DI water, acetone for 15 min by ultrasonic bath (the water and acetone repeated for three times during washing), respectively. The solid was then dried under vacuum at 60 ℃.

Fabrication of Micro-supercapacitors Based on Co-BTA: First, 150 nm Au interdigital electrode was evaporated on the silicon wafer. Then the brown film obtained at the air-liquid interface was transferred onto the Au patterned substrate by immersing the substrate into the solution. Then, the H_2SO_4-polyvinyl alcohol (H_2SO_4-PVA) gel electrolyte was cast on the Co-BTA-Au:SiO_2 substrate. Finally, the device was left to solidify overnight to form a solid-state Co-BTA-Au MSC.

Material characterization : Scanning electron microscopy images were taken by using a Jsm-7800F scanning electron microscope. X-ray diffraction patterns of out-of-plane and in-plane were obtained by using Smartlab (3KW) and Smartlab Ⅲ, respectively. Reflex module implemented in Material studio 8.0 (Accelrys Inc.) was employed to simulate the Co-BTA XRD pattern. UV-visible absorption spectra was acquired from Shimadzu UV-1750 at room temperature. Phi 5000 VersaProbe was used to record the X-ray photoelectron spectroscopy images. Tensor 27 was used to obtain IR spectra. Cyclic voltammograms of product and ferrocene were gained by using CHI 660D electrochemical workstation with a scanning rate of 100 mV/s.

Electrochemical measurements: Cyclic voltammetry (CV) and electrochemical impedance spectroscopy (EIS) were performed by CHI 660D electrochemical workstation (Chenhua). The CV was tested with the scan rate ranging from 0.05 to 1000 V s^{-1}. EIS was recorded in the

frequency range of 1 Hz to 100 kHz with a 5 mV ac amplitude. The H_2SO_4- PVA gel electrolyte was prepared by mixing 1 g H_2SO_4 and 1 g PVA in 10 mL deionized water and heated up to 60 °C for 6 h with a stirring rate of 800 rpm. The capacitance values of the device were calculated from the CV data by equation (1):

$$C_{device} = \frac{1}{v \times (V_f - V_i)} \int_{V_i}^{V_f} I(V)dV$$

(1)

the C_{device} is used as the capacitance contribution from Co-BTA film electrodes; v is the scan rate (in V s^{-1}); V_f and V_i are the intergration potential limits of the voltammetric curve and $I(V)$ is the voltammetric discharge current (in amperes); $\int_{V_i}^{V_f} I(V)dV$ is the integrated area from the CV curves. The total surface area of the device including the spacing between the electrodes was 0.6 cm^2, and the thickness of the active materials are 60 nm. This is used to calculate the power and energy density.

The volumetric (C_V in F cm^{-3}) capacitance was calculated by equation (2):

$$C_V = \frac{C_{device}}{V} \tag{2}$$

V is the volume of the device, respectively.

The volumetric (E_V in Wh cm^{-3}) energy densities was calculated from the equation (3):

$$E_V = \frac{1}{2} \times C_V \times \frac{(\Delta V)^2}{3600}$$

(3)

the ΔV is the potential range (in volts).

The volumetric (P_V in W cm^{-3}) power densities was calculated by equation (4):

$$P_V = \frac{E_V}{\Delta t} \times 3600$$ (4)

Δt is the discharge time in seconds.

The EIS was measured to investigate the AC-line filtering performance of the micro-supercapacitors based on Co-BTA film. The specific capacitance of the microdevice can be described by $C'(f)$ and $C''(f)$ on the basis of the equation (5) and (6):

$$C^{'}(f) = \frac{-Z''(f)}{2\pi f S|Z(f)|^2}$$

(5)

$$C^{'}{}'(f) = \frac{Z'(f)}{2\pi f S|Z(f)|^2}$$

(6)

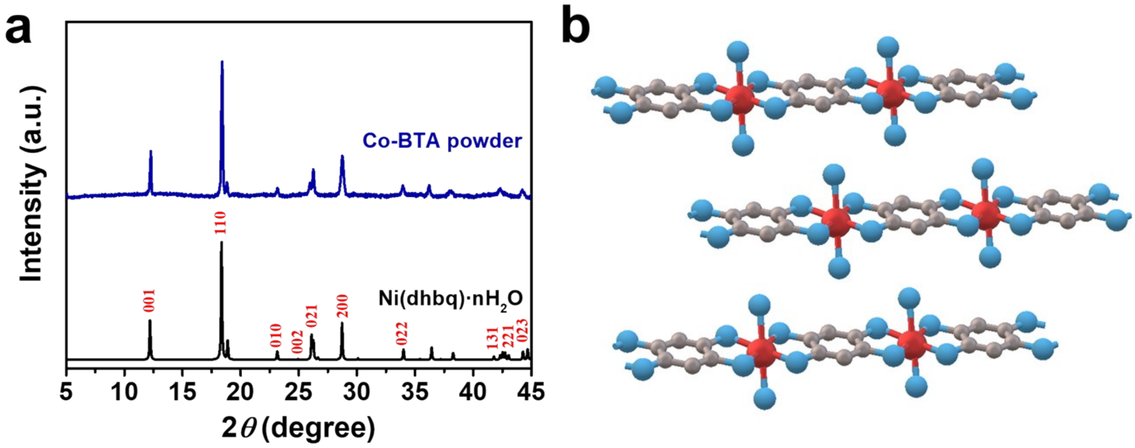

Figure S1. (a) PXRD profile of Co-BTA powder and simulated PXRD pattern of Ni(dhbq)•nH_2O.[1] (b) Simulated crystal structure of Ni(dhbq)•nH_2O.[1] Red, blue and grey balls correspond to nickel, oxygen and carbon atoms, respectively. Hydrogen atoms are deleted in the crystal structure for a simplified view.

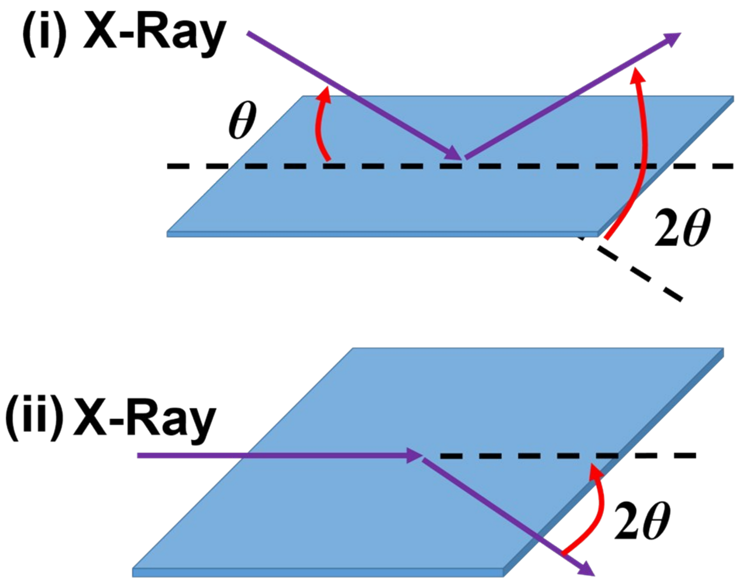

Figure S2. The schemes of the orientation of the films for (i) out-of-plane XRD scan and (ii) in-plane XRD scan.

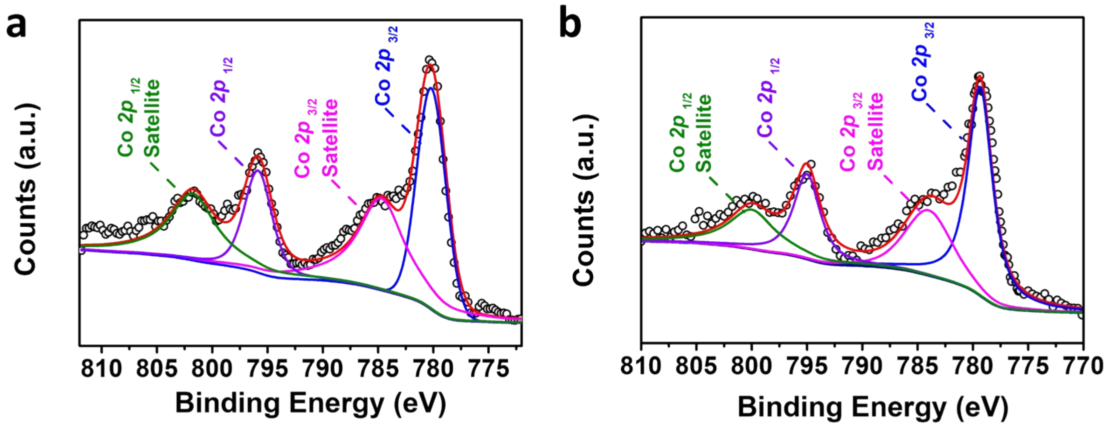

Figure S3. Co 2p core level spectra of (a) Co-BTA film and (b) Co-BTA powder. The Co 2p spectra of XPS could be fitted into two sets of doublets, among which the peak at ~780 eV together with the satellite peak at ~785 eV corresponds to Co 2p3/2 and the peak at ~795 eV togeter with the satellite peak at ~801 eV belongs to the Co 2p1/2, respectively.

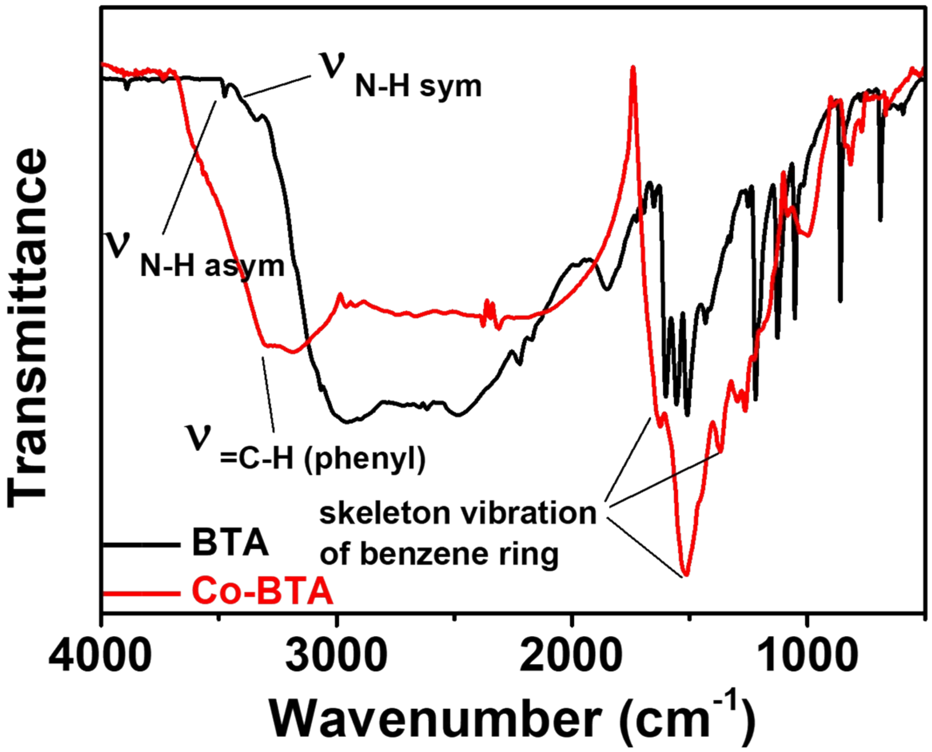

Figure S4. IR spectra of the Co-BTA film and BTA ligand.

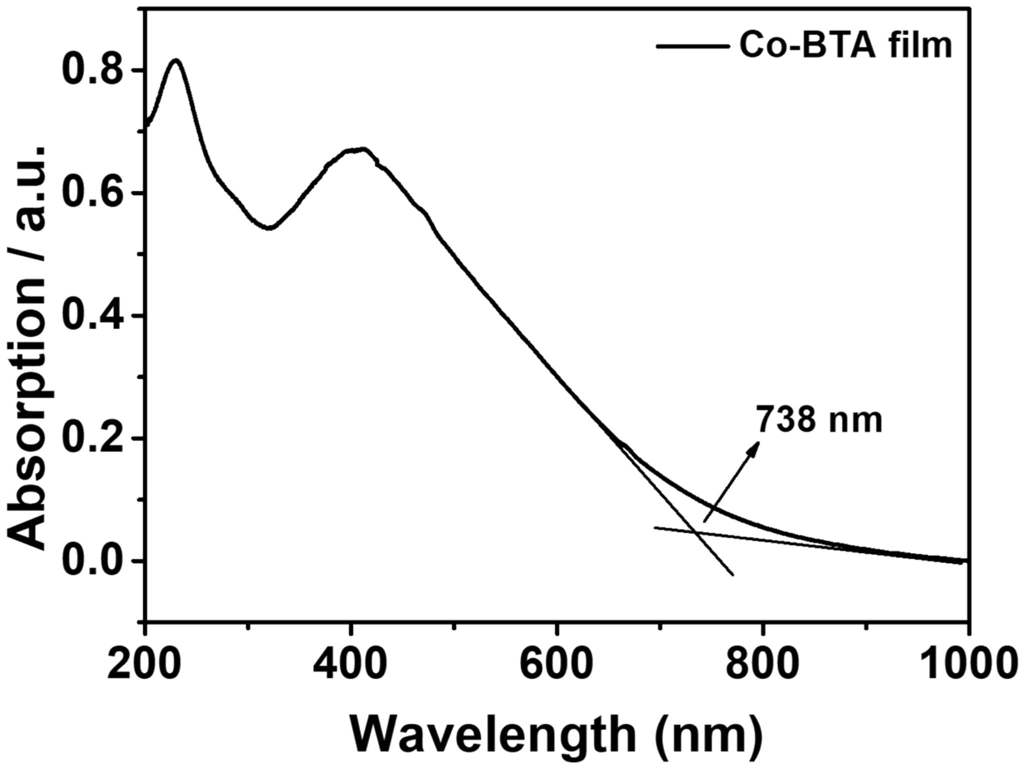

Figure S5. The normalized UV-Vis spectra of the Co-BTA film.

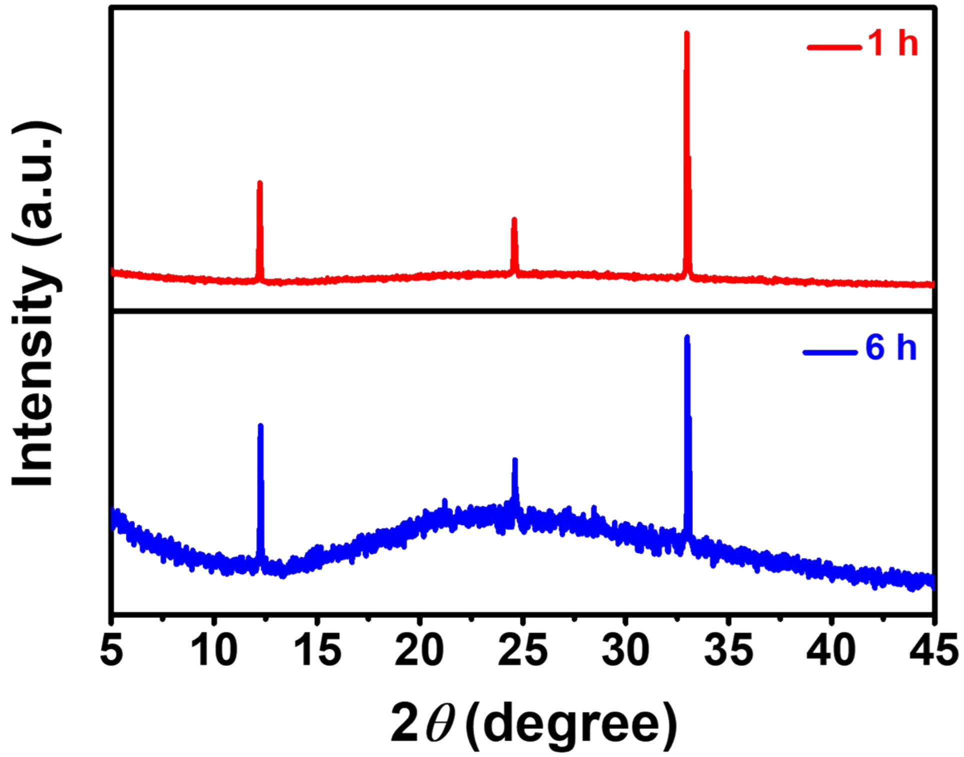

Figure S6. Out-of-plane PXRD profiles of Co-BTA film after immersed into the acid solution for 1 hour and 6 hour, respectively.

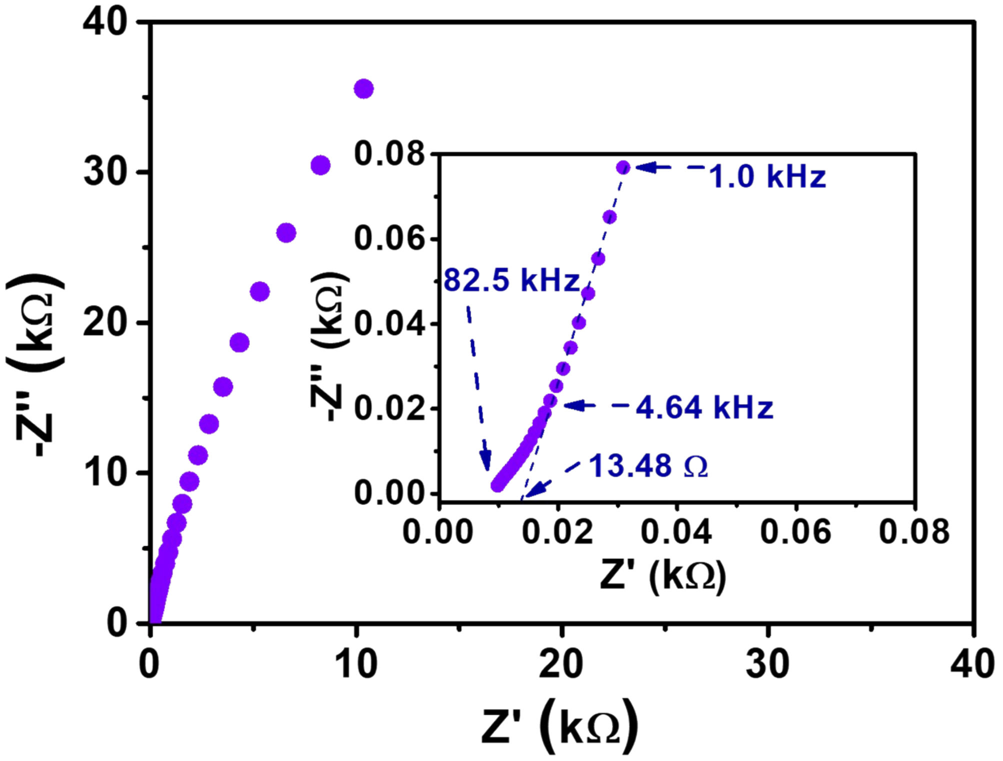

Figure S7. Complex plane plot of the impedance of the Co-BTA-based microdevices. Inset displays a magnification of the high-frequency region.

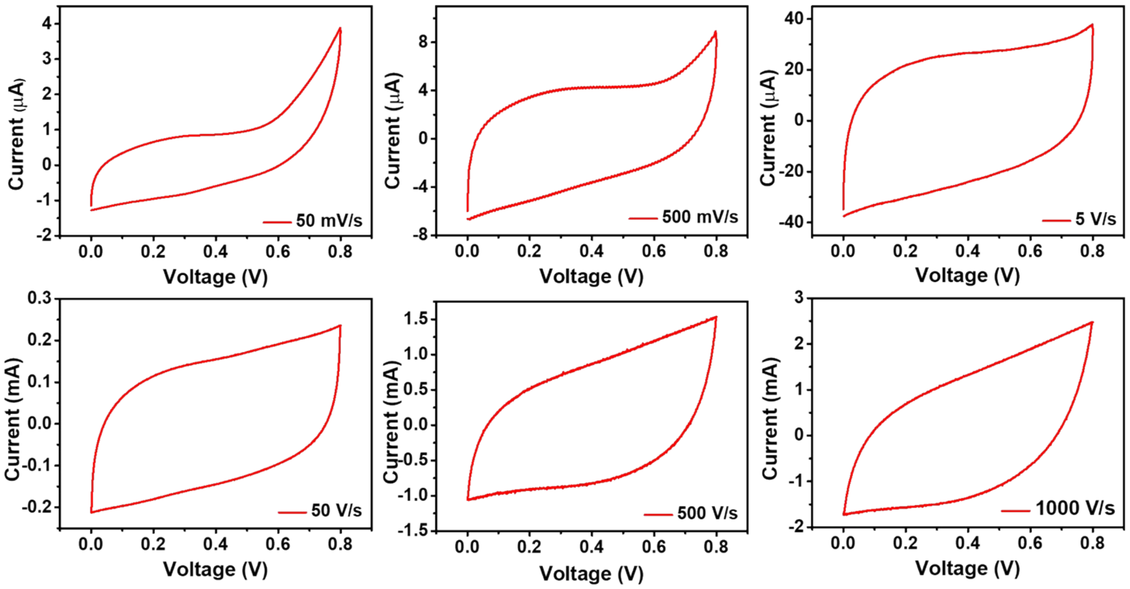

Figure S8. CV curves of Co-BTA-based MSC with a PET substrate in the H_2SO_4-PVA gel electrolyte at different scan rates.

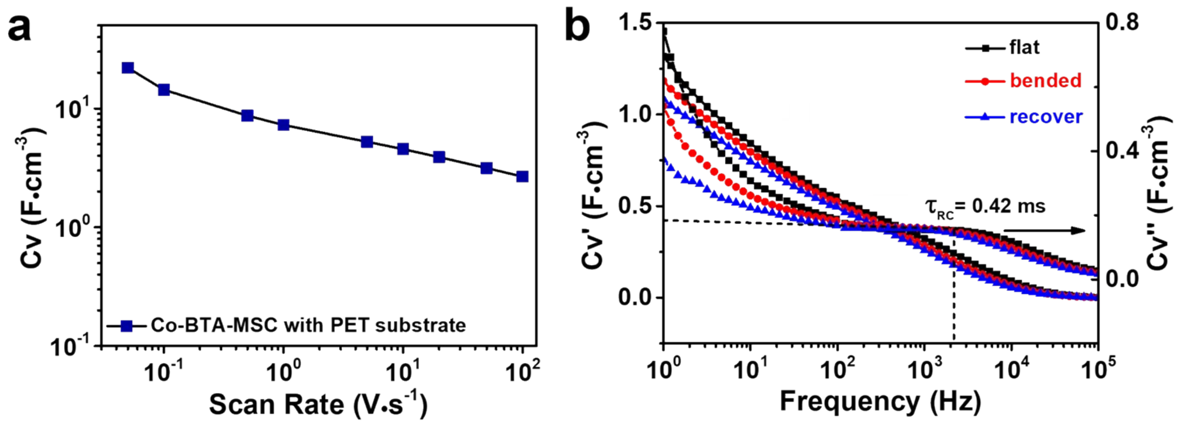

Figure S9. Electrochemical performance of Co-BTA-based-MSC with flexible PET substrate. (a) CV evolution of the as-fabricated flexible MSC before bending test. (b) Plot of capacitance (CV'=volumetric real capacitance and CV''=imaginary capacitance) versus the frequency of flexible MSC in different states (flat, bended, and recover), bending radius = 1cm.

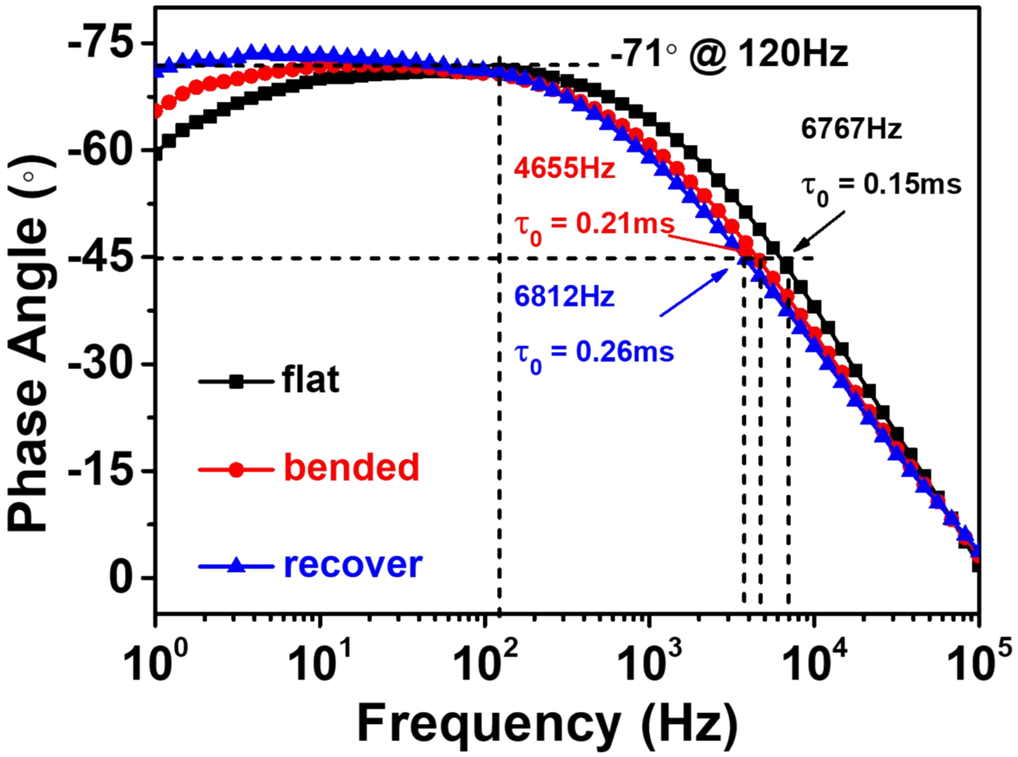

Figure S10. Impedance phase angle on the frequency of Co-BTA-based flexible microdevices in different states (flat, bended and recover), bending radius = 1cm.

Table S1. Element quantities analysis of C, O, N, Co of Co-BTA film and Co-BTA powder based on the XPS analysis.

Co-BTA	Element	C	O	N	Co
Film	At%	56.06	23.64	15.80	4.50
Powder	At%	60.75	21.23	14.20	3.82

Table S2. The performance of the reported micro-supercapacitors based on different materials.

Material	E[a]	VW[b]	Cv[c]	Ev[d]	Pv[e]	angle[f]	f_0[g]	τ_0[h]	τ_{RC}[i]	Ref
Co-BTA	PVA-H_2SO_4	0-0.8	23 at 50 mV s^{-1}	1.6 at 50 mV s^{-1}	1056 at 1000 V s^{-1}	-78.6°	6812	0.15	0.32	**This Work**
Onion-like Carbon	$TEABF_4$-PC	0-3.0	1.35 at 1 V s^{-1}	1.6 at 1 V s^{-1}	250 at 200 V s^{-1}	-	<100	<10	26	**2**
Carbon Nanotubes/ Graphene	1M Na_2SO_4	0-1.0	1.1 at 1.0 A cm^{-3}	0.16	115	-81.5°	1343	0.82	0.195	**3**
PiCBA	PVA-H_2SO_4	0-1.0	34.1 at 50 mV s^{-1}	4.7 at 50 mV s^{-1}	1323 at 1000 V s^{-1}	-73°	3620	0.27	0.83	**4**
inkjet-printed EC	1M Et_4NBF_4	0-2.5	-	-	-	-	< 5	< 200	-	**5**
UPSCs-25	PVA-H_2SO_4	0-1.0	348	12 at 10 mV s^{-1}	4386 at 2000 V s^{-1}	-72°	1000	1	0.47	**6**
vertically oriented graphene	25% KOH	-	3	-	-	-82°	15000	0.067	0.2	**7**
Sulfur-Doped Graphene	PVA-H_2SO_4	0-1.0	582	3.1 at 10 mV s^{-1}	1191 at 6000 V s^{-1}	-73°	3836	0.26	-	**8**
Carbon Nanotubes/ Reduced Graphene Oxide	3M KCl	0-1.0	3.1 at 10 mV s^{-1}	0.68 at 1 V s^{-1}	77 at 50 V s^{-1}	-	208.6	4.8	-	**9**
EG/PH1000	PVA-H_2SO_4	0-1.0	27			-84°	4200	0.24	1.35	**10**
MPG-MSCs	PVA-H_2SO_4	0-1.0	17.9	2.5 at 0.01 V s^{-1}	495 at 1000 V s^{-1}	-	3579	0.28	-	**11**

a) electrolyte; b) voltage window (V); c) specific volumetric capacitance (F cm^{-3}); d) energy density (mWh cm^{-3}); e) power density (W·cm^{-3}); f) phase angle @120 Hz; g) characteristic

frequency (Hz); [h)] relaxation time (ms); [i)] resistance-capacitance time (ms).

References

1 T. Yamada, S. Morikawa and H. Kitagawa, *Bull. Chem. Soc. Jpn.*, 2010, **83**, 42-48.

2 D. Pech, M. Brunet, H. Durou, P. Huang, V. Mochalin, Y. Gogotsi, P. Taberna and P. Simon, *Nat. Nanotechnol.*, 2010, **5**, 651-654.

3 J. Lin, C. Zhang, Z. Yan, Y. Zhu, Z. Peng, R. H. Hauge, D. Natelson and J. M. Tour, *Nano. Lett.*, 2012, **13**, 72-78.

4 C. Yang, K. S. Schellhammer, F. Ortmann, S. Sun, R. Dong, M. Karakus, Z. Mics, M. Löffler, F. Zhang, X. Zhuang, E. Cánovas, G. Cuniberti, M. Bonn and X. Feng, *Angew. Chem. Int. Ed.*, 2017, **56**, 3920-3924.

5 D. Pech, M. Brunet, P. Taberna, P. Simon, N. Fabre, F. Mesnilgrente, V. Conédéra and H. Durou, *J. Power Sources*, 2010, **195**, 1266-1269.

6 Z. Wu, Z. Liu, K. Parvez, X. Feng and K. Müllen, *Adv. Mater.*, 2015, **27**, 3669-3675.

7 J. R. Miller , R. A. Outlaw and B. C. Holloway , *Science*, 2010, **329**, 1637-1639 .

8 Z. Wu, Y. Tan, S. Zheng, S. Wang, K. Parvez, J. Qin, X. Shi, C. Sun, X. Bao, X. Feng and K. Müllen, *J. Am. Chem. Soc.*, 2017, **139**, 4506-4512.

9 M. Beidaghi and C. Wang, *Adv. Funct. Mater.*, 2012, **22**, 4501-4510.

10 Z. Liu, Z. Wu, S. Yang, R. Dong, X. Feng and K. Müllen, *Adv. Mater.*, 2016, **28**, 2217-2222.

11 Z. S. Wu, K. Parvez, X. Feng and K. Müllen, *Nat. Commun.*, 2013, **4**, 2487-2494.
